# *Streptococcus equi* subsp. *equi* in Retropharyngeal Abscess: Case Report and Review of Literature

**DOI:** 10.3390/microorganisms10102032

**Published:** 2022-10-14

**Authors:** Anna Waśniewska-Włodarczyk, Renata Pepaś, Renata Janowicz, Wiesław Konopka

**Affiliations:** 1 Department of Otolaryngology, Polish Mother’s Memorial Hospital Research Institute, 93-338 Lodz, Poland; 2 Department of Normal and Clinical Anatomy, Medical University of Lodz, 90-419 Lodz, Poland; 3 Department of Paediatric Didactics, Medical University of Lodz, 90-419 Lodz, Poland

**Keywords:** retropharyngeal abscess, laryngology, zoonosis, *Streptococcus equi*

## Abstract

Retropharyngeal abscesses (RPAs) represent the group of deep space infections of the neck. Although RPA is a well-known condition, some aspects of it still may be challenging. Localization, symptoms, and etiology may confuse even the most experienced physicians. *S. equi* subspecies are zoonotic agents and cause multiple diseases in diverse animals. Infections in humans are rare. This report presents an extremely rare case of retropharyngeal abscess in a 12-year-old girl caused by an infection of *Streptococcus equi* subsp. *equi.*

## 1. Introduction

Retropharyngeal abscesses (RPAs) represent the group of deep space infections of the neck. RPAs constitute up to 22% of all infections in this area [1]. Nowadays, due to antibiotics development, RPAs are a rare condition, but however are associated with serious, even fatal complications [2,3]. The most exposed group of patients are children from three to five years old [4]. The retropharyngeal space is a potential space, posteriorly limited by the prevertebral fascia, that extends from the skull base to the pharynx in the mediastinum, the potential pathway to the chest. The constrictor muscles of the pharynx are anterior to the retropharyngeal space, and laterally are the carotid sheaths. The space contains multiple lymphatic nodes, that drain lymph from tissues in the nose and sinuses, Eustachian tubes down to the surrounding pharyngeal tissues [1,4]. Symptoms of RPAs are usually nonspecific, and may occur as: odynophagia, dysphagia, fever, neck stiffness or neck swelling, lymphadenopathy, drooling [5]. Due to serious complications RPAs should be early diagnosed. RPAs may be differentiated with cellulitis or lymphadenopathy, tonsilitis, peritonsilar abscess, retropharyngeal calcific tendinintis. RPAs are usually caused by multiple microorganisms, mostly by aerobes (*Streptococcus viridans*, group A *Streptococcus*, *Staphylococcus aureus, Sepidermidis*) and anaerobes *(Fusobacterium*, *Bacteroides*, *Peptostreptococcus* sp.) [4]. Most cases of RPAs are treated conservatively with ampicillin/sulbactum, clindamycin, cephalosporins and metronidazole. Surgical approach is used in the case of larger abscesses [6,7].

This report presents an extremely rare case of retropharyngeal abscess in a 12-year-old girl caused by an infection of *Streptococcus equi* subsp. *equi*.

## 2. Case Report

**Figure 1:**
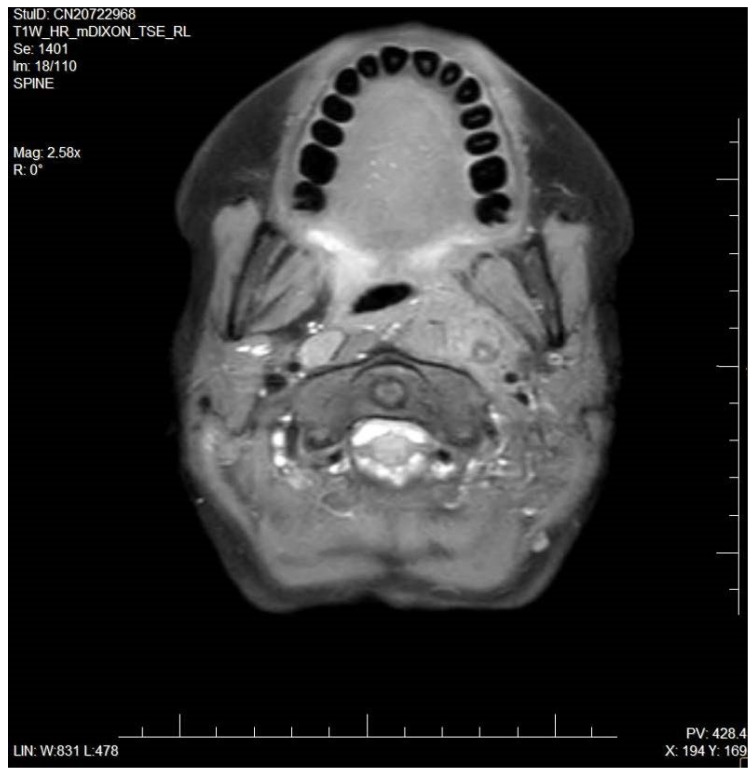
Magnetic resonance of head-transverse plane.

**Figure 2:**
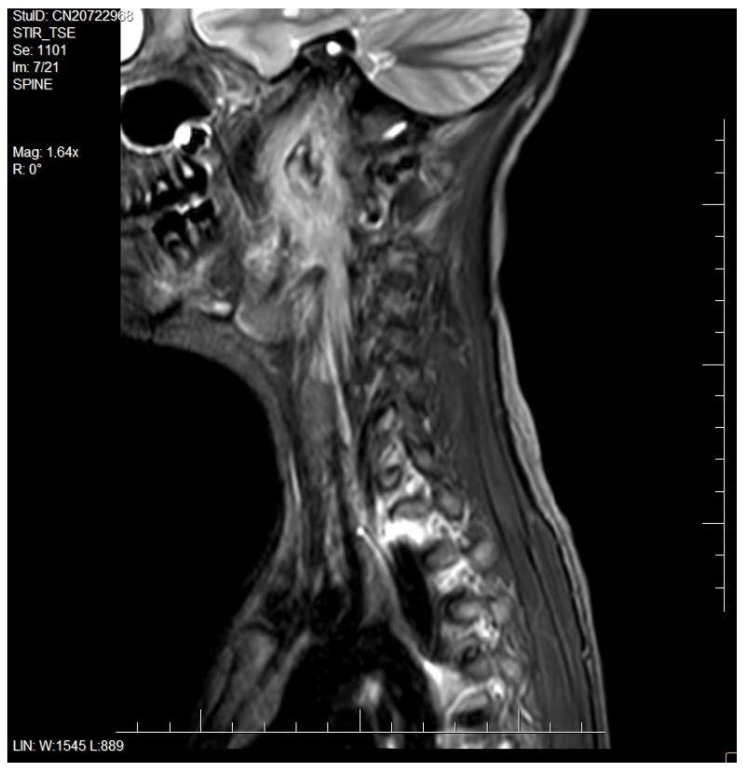
Magnetic resonance of head-saggital plane.

A 12-year-old girl was presented to the Department of Pediatric Otorhinolaryngology with severe neck pain and stiffness, odynophagia, and a sore throat. The girl was admitted with a hospital referral form from her family doctor with suspicion of peritonsillar abscess. Three weeks before symptoms occurred the patient had an experience of pharyngitis with fever. During examination at the hospital, the otolaryngologist noticed an asymmetrical prominence of the left palatino-lingual arch, left palatine tonsil enlargement, and pain during neck palpation. Her C-reactive protein (CRP) level was mildly raised (13.61 mg/L), and she demonstrated significant leukocytosis (19,650 cells/μL) and neutrophilia (16,330 cells/μL). The patient was also tested for an infection of Ebstein–Barr virus, and received a positive result of: IgG Anti-VCA (363 U/mL) and IgG EBV Nuclear Antigen (>600 U/mL), while the IgM EBV antibodies result was negative. She was commenced on intravenous rehydration and cefuroxime. After consultation with a radiologist, an otolaryngologist recommended ultrasonography (USG) and magnetic resonance (MR) of the neck (Figure 1 and Figure 2). USG revealed an enlargement of lymph nodes, up to 6 mm, with edema of fat tissue on the left side of the neck. MR revealed soft tissue infiltration with dimensions of 40 mm × 35 mm × 90 mm on the left side of the retropharyngeal space. Infiltration displaced the left torus of the auditory tube and soft tissue of the left side of the pharynx to the left palatine tonsil. An abscess with dimensions 15 mm × 10 mm × 40 mm was located in the center of infiltration. Lymph nodes of the neck were enlarged up to 12 mm. Infiltration penetrated through the deep tissues of the neck and surrounded the internal jugular artery. The patient received additional intravenous antibiotic-amikacin and was qualified for surgery. During surgery, the abscess was cut and drained. Collected purulent content was submitted for microbiological examination. The girl received other intravenous and oral medicines, vancomycin, fluconazole, and metamizole. On the fourth postoperative post-operating day, MR was repeated. The dimensions of the abscess were decreased to 30 mm × 8 mm × 10 mm; moreover, space of infiltration was slightly lesser. The patient was reoperated, and the wound was broadened and drained from bloody content. After surgery, the comfort of the patient was improving and she recovered full movement potential of neck. Results of microbiological examination revealed three other types of bacteria: *Streptococcus equi* subsp. *equi*, *Streptococcus mitis*, *Sphingomonas paucimobilis*. Pathogens were susceptible to administered antibiotics. The patient was discharged from the hospital in good general and local condition.

## 3. Discussion

Usually, RPAs develop as a complication of upper respiratory tract infection such as pharyngitis, lymphadenitis, or tonsillitis. Sometimes RPA is idiopathic, but in other cases it may be a result of trauma, foreign body ingestion, or immunodeficiency [4]. RPAs are frequently caused by mixed flora [8,9], mostly with predominant pathological conversion of commensals common in the upper respiratory tract and also usual offending pathogens such as *Streptococcus viridians* or *Staphylococcus epidermidis* and aureus, with other anaerobes, Gram-positive bacteria or viruses also seen in [1,9,10]. However, sometimes pathogens are unusual as in this case. *Streptococcus equi* subsp. *equi* belong to the C group *Streptococci* [11]. This group is also represented by two other pathogens: *S. equi* subsp. *zooepidemicus* and *S. equi* subsp. *ruminatorum* [12]. All subspecies of *S. equi* are zoonotic agents and cause multiple diseases in diverse animals [13]. *S. equi* subsp *ruminatorum* causes an inflammation of the mammary gland of small ruminant flocks and severe infections in zebras and hyenas [14,15]. To date, there have been only a few case reports regarding the zoonosis of this pathogen in humans. In 2007 Marchandin et al. reported a case of a 53-year-old man infected with HIV and later *S. equi* subsp. *ruminatorum* [13]. Infection caused severe inflammation of the respiratory tract and sepsis which resulted in brain death. Further, in 2011 Meyer et al. described a case of a 70-year-old man, who had occasional contact with horses. The patient was diagnosed with acute endocarditis, which involved the anterior mitral valve and spondylodiscitis [16]. Also in 2012, as a consequence of infection with *S. equi* subsp *ruminatorum*, a 63-year-old man was diagnosed with prosthetic aortic valve endocarditis, he also had occasional contact with horses [17]. *S. equi* subsp. *zooepidemicus* is a commensal bacterium of horses’ airways and may also affect wound, uterine, and respiratory infections in horses [17]. Transmission from animals to humans is a sparseness, however, it is more common in comparison with *S. equi* subsp. *ruminatorum* [18,19]. *S. equi* subsp. *zooepidemicus* in humans may lead to bacteriemia, meningitis, spondylodiskitis, pneumonia, septic arthritis, toxic shock-like syndrome, nephritis, and endocarditis [18,19,20,21,22,23,24]. *S. equi* subsp. *equi* causes strangles in equine, which is characterized by lymphadenitis of the upper respiratory tract and pharyngitis [25]. Strangles is a common but severe disease in horses [25]. In humans, *S. equi* subsp. *equi* provokes acute infections in immunocompromised hosts, usually after close contact with horses. Unfortunately, these infections are associated with high mortality [26]. Four cases reported inflammation of the central nervous system. In 2003, Elsayed et al. reported a 13-year-old boy who lived on a farm with horses. He suffered from a headache, fever, neck stiffness, vomiting, nausea, anorexia, photophobia, ataxia, and bilateral deafness. The boy was diagnosed with bacterial meningitis and received antibiotic treatment. The patient recovered with mild residual ataxia and sensorineural hearing loss for which he received a bilateral cochlear implant [27]. Popescu et al. reported a case of a 75-year-old female, whose neighbors were horse owners and she was visiting them two weeks before admission to the hospital [28]. The woman was admitted with a fever and stupor. The patient was successfully treated with antibiotics and glucocorticosteroids. Also a 13-year-old-boy with systemic lupus erythematosus, after contact with an infected pony, developed meningitis and sepsis [26]. The patient recovered fully after eight weeks of intravenous antibiotics. Another infection of the central nervous system was described by Kerstens et al. They reported a case of a 69-year-old man, who returned a month earlier from vacation in Myanmar that included horseback riding [29]. The man was admitted to the hospital with confusion, agitation, shivering, and incontinence of urine. Two days before admission, he complained about headache, pain of the left ear, and night sweats. The patient was treated with antibiotics, however, after 5 months he was back in the hospital due to complications—superficial dural arteriovenous fistula. In 2016, Brzezinski and Chiriac reported a case of a 38-year-old man, who worked in a horse stable [30]. The patient suffered from the skin lesions: multiple and confluent, erythematous, crusted plaques over the face, chin, and neck. He had subcutaneous abscesses, enlarged lymph nodes, limitation of mobility of the neck and dysphagia. Infection was caused by group C *Streptococci* (probably *Streptococcus equi*). The patient recovered after receiving antibiotics for two weeks. 

To the best of our knowledge, it is the first case describing retropharyngeal abscess caused by *S. equi* subsp. *equi* in a child. The patient was exposed to this rare bacterial infection probably during her horse riding lessons. However, the patient’s legal guardian denied that patient could have any contact with a sick horse. Moreover, horses from the stable were under the care of qualified veterinarians. All horses should be examined by veterinarians before and after transport to decrease the risk of disease transmission [31]. According to Newton et al. [32], horses, that have recovered from infection caused by *S. equi*, even though they do not present any symptoms, are still a carrier of the pathogen. Unfortunately, veterinary examinations identify animals suffering from acute disease, which may initiate transmission to new populations of horses. There is no immunodeficiency history in this patient. Predisposing factor to this infection was probably a preceding infection of EBV, whichthe patient suffered from a couple of weeks before symptoms of abscess. 

## 4. Conclusions

Although symptoms, diagnosis, etiology, and treatment of RPAs are well-described in the literature, there are still some aspects that may surprise us. The symptoms of RPA are ambiguous, thus diagnosis of RPA, may be challenging, and clinicians must keep it in their differential diagnosis. As shown in the above case, a microbiological examination is crucial in appropriate treatment. To avoid zoonosis infections, patients should be more aware of the importance of hygiene (frequent hand washing, changing clothes after contact with animals, avoiding bites and scratches from animals) and the accuracy of veterinarian examinations should be improved.

## Data Availability

Not applicable.
